# Reorienting Oral Health Services to Prevention: Economic Perspectives

**DOI:** 10.1177/0022034520986794

**Published:** 2021-01-21

**Authors:** C.R. Vernazza, S. Birch, N.B. Pitts

**Affiliations:** 1 School of Dental Sciences, Newcastle University, Newcastle Upon Tyne, UK; 2 Centre for the Business and Economics of Health, University of Queensland, Saint Lucia, Queensland, Australia; 3 Centre for Health Economics, University of Manchester, Manchester, UK; 4 Faculty of Dentistry, Oral and Craniofacial Sciences, Kings College London, London, UK

**Keywords:** caries detection/diagnosis, decision making, dental public health, economic evaluation, periodontal disease(s)/periodontitis, preventive dentistry

## Abstract

Despite the recognized need to change the emphasis of health services by shifting the balance from treatment to prevention, limited progress has been made in many settings. This is true in oral health, where evidence for preventive interventions that work has not been systematically exploited in oral health services. While reorienting health services is complex and context specific, economics can bring a helpful perspective in understanding and predicting the impact of changes in resource allocation, provider remuneration systems, and patient payments. There is an increasing literature on the economics of different prevention approaches. However, much of this literature focuses on the costs and potential savings of alternative approaches and fails to take into account benefits. Even where benefits are taken into account, these tend to be narrowly focused on clinical outcomes using cost-effectiveness analysis, which may be of little relevance to the policy maker, patient, and the public. Some commonly used economic approaches (such as quality-adjusted life years and incremental cost-effectiveness ratios) may also not be appropriate to oral health. Using alternative techniques, including wider measures of benefit and employing priority setting and resource allocation tools, may provide more comprehensive information on economic impact to decision makers and stakeholders. In addition, it is important to consider the effects of provider remuneration in reorienting services. While there is some evidence about traditional models of remuneration (fee for service and capitation), less is known about pay for performance and blended systems. This article outlines areas in which economics can offer an insight into reorientation of health systems toward prevention, highlighting areas for further research and consideration.

## Introduction

Oral diseases are some of the most prevalent diseases globally. The 2017 Global Burden of Diseases (GBD) study estimated 3.5 billion people were affected by oral diseases with a loss of 15 million disability-adjusted life years (Bernabe et al. 2020). The global costs of these diseases were estimated from earlier GBD studies at US$298 billion directly for treatment and US$144 billion in productivity losses (Listl et al. 2015). Caries and periodontal disease form most of this burden, and in order to limit the scope of this article, only these 2 diseases will be considered.

While the global burden remains high, after population size and age profile adjustment, this has decreased for caries over the past 30 y while increasing for periodontal disease. However, these changes vary in different countries and in World Bank Income Groups of countries (Bernabe et al. 2020). In order to reduce prevalence and burden, there has been a growing call for an increase in oral disease prevention (e.g., Pitts and Zero 2016) fitting with the agenda across health more generally (World Health Organization 2013).

It is apparent that where reductions in prevalence of oral disease have occurred, oral health services (i.e., dental professionals providing dental care to individuals in clinical settings) are responsible for only a very small proportion of any decrease in prevalence, with population-based measures and wider social determinants playing a greater role (Sheiham 1997). While it is difficult to quantify the relative importance of individual and population-based measures, some individual-level approaches have been criticized as being ineffective or found to increase inequalities (Kay and Locker 1996). It is likely that wider social and commercial determinants will continue to be the key aspects that must be addressed, yet oral health services do have an important role to play in oral disease prevention (Watt et al. 2019). Historically, oral health services have focused on treatment rather than prevention and while some progress has been made in reorienting these services toward prevention, a treatment focus persists driven by a combination of a limited understanding of disease pathogenesis, a surgical approach to care, and high prevalence of active disease (Steele 2009).

**Figure 1.**
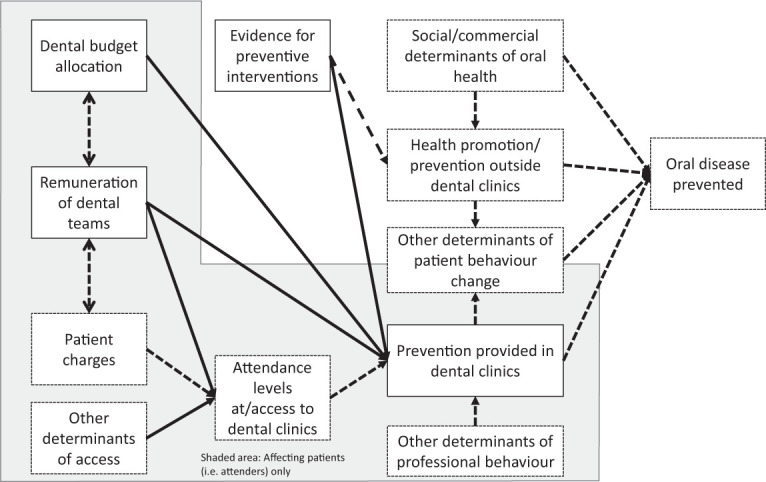
Model to show economic influences on prevention of oral disease and the scope of this article (solid lined areas are covered with dashed areas not covered in the article).

Reorienting oral health services is a complex area (Birch et al. 2015), but economics offers a valuable perspective. A model of the potential influences on provision of preventive care and oral disease prevented is shown in Figure 1, and the limits of what is explored in this article are noted, acknowledging that the other areas also warrant further exploration. In particular, it is important to acknowledge the complex interaction between changes in social determinants and population health measures and oral health services, but exploration of these is beyond the scope of this review.

We therefore explore issues in reorienting oral health services toward disease prevention from an economic perspective, particularly from the perspective of a policy maker responsible for making decisions about the provision of individual-level services. First, we discuss whether there is evidence for interventions that work before turning to current issues of resource allocation and provider remuneration. The use of economic techniques to provide guidance and predictions on the impact of reorientation toward prevention is then outlined, followed by an overview of empirical evidence on the impacts of such reorientation. The arguments presented were developed from 2 “dental policy labs” hosted by the Alliance for a Cavity Free Future and Kings College London (Pitts et al. 2017; Pitts et al. 2019).

## Is There Sufficient Evidence for Prevention in Oral Health?

When considering why oral health services are not oriented to prevention, it is important to explore whether there is evidence about efficacy and effectiveness of individual-level measures in preventing caries and periodontal disease.

For caries, primary prevention relies on exposure to fluoride, reduction of free sugars in the diet, oral hygiene, and alteration of the oral microflora (Twetman 2018). Many of these can be addressed through population-based schemes such as sugar taxation; fluoridation of water, milk, and salt; and community-based toothbrushing schemes. However, these fall outside the remit of this review. There is substantial evidence of the preventive effect of individual-level interventions that address these factors, including fluoridated toothpaste (Walsh et al. 2019), fluoride varnish (Marinho et al. 2013), and fissure sealants (Ahovuo-Saloranta et al. 2017). While use of some of these measures depends on individual behaviors, health services may have a role in education and motivation. Secondary and tertiary prevention of caries relies on early detection and management of initial lesions, including minimally interventive techniques. Various evidence-based guidelines are available, including the widely accepted International Caries Classification and Management System ICCMS (Pitts et al. 2013). As the breadth of primary, secondary, and tertiary prevention is frequently not appreciated by decision makers, current international consensus on terminology is now to refer to caries *care*, or *management* or *control* (Machiulskiene et al. 2020).

For periodontal disease, primary prevention mainly relies on oral hygiene measures and chemical controls to reduce and disrupt the biofilm, principally through the use of additives to toothpastes and mouthrinses, as well as control of tobacco (in the individual-level setting through smoking cessation programs) (Herrera et al. 2018). The evidence for the effect of toothbrushing (Yaacob et al. 2014) and chemical agents (Serrano et al. 2015) is substantial, although the evidence for interdental aids is less robust (Worthington et al. 2019). In the area of tobacco control, there is a large body of evidence on effective interventions (Beaglehole and Benzian 2005; Lindson-Hawley et al. 2018). Secondary and tertiary prevention of periodontal disease again relies on early diagnosis and appropriate management, with evidence underpinning many different approaches (Graziani et al. 2017).

## Current Levels of Prevention in Oral Health Services

There are very limited data on the proportions of dental budgets spent on different aspects of care. However, estimates for the OECD (Organisation for Economic Co-operation and Development) countries suggest that spending on prevention across all health care represents less than 3% of total health care expenditure (Gmeinder et al. 2017). In lower-income countries, while figures for all health care are not available, the proportion of *primary* health care spend on prevention is higher than high-income countries, but the absolute spend is very low (US$26/capita/annum compared to US$1,303, respectively) (World Health Organization 2019).

While the nature of oral health services differs between countries, some broad conclusions can be drawn about current levels of prevention, based on a 2018 World Health Organization survey (Petersen et al. 2020). This survey (Petersen et al. 2020) reported that for preschool-aged children, 43% of low-income countries offered dental examinations (although the financial basis on which it was “offered” was not made clear), compared to 67% and 66% of middle- and high-income countries, respectively. For adults, examinations were provided in 29%, 36%, and 54% of low-, middle-, and high-income countries, respectively, although again the patient financial arrangement was not clear. Across all types of countries, fluoride varnish was offered to adults in only 20% of countries, although figures were higher for children. While the preventive effect of dental examinations (at all levels of prevention) is unclear and definitions may vary (Fee et al. 2020), this measure nonetheless gives an insight into the provision of preventively orientated services in different countries.

## The Economic Issues in Reorienting Oral Health Services

Given the substantial evidence for effectiveness of interventions preventing caries and periodontal disease but limited progress with implementation, we identify 3 main economic barriers to reorientation of oral health services toward prevention:

1. Scarce resources for oral health services
2. Methods of remuneration of dental teams that appropriately incentivize prevention
3. Access to dental services in populations, including the affordability of out-of-pocket costs

While we are unable to cover the third barrier within this article, it is important to remember that this will have an impact on both of the other issues discussed here, each of which will now be considered in turn.

### The Allocation of Scarce Resources

There are never sufficient health care resources (e.g., staff, estate, equipment) to do everything that we would like to do (the principle of scarcity), leading to a need for prioritization. Although many have called for increased resources for prevention, investing further in this area implies that something else must be forgone (the principle of opportunity cost), even if new money is made available, as real resources (e.g., personnel or estate) will still be constrained.

If, for example, a hypothetical dental budget is to remain stable (i.e., increasing only in line with inflation) (a common scenario since the global financial crisis of 2010; Ortiz et al. 2015), increasing the amount spent on prevention would mean less spent on treatment. The same treatment might still be delivered if this can be done more efficiently (i.e., producing the same output for less resource input), but where efficiencies cannot be realized, the benefit of using the resources for prevention would have to be weighed against the benefit of continuing to use the resources for treatment. Often the difficulty in making these decisions is that the “benefit” encompasses various different things. Some may think of benefit in clinical terms (reducing caries incidence, for example), whereas others may think that other objectives, such as reducing social inequalities in health or increasing productivity of the workforce, are more important, or “benefit” may be seen most broadly as societal well-being. In making allocation decisions, usually a broader measure will be more appropriate, but this is often not explicitly defined. Dentistry sometimes faces additional difficulties in that some interventions may be focused on achieving the improvement of appearance, which some may argue are not a core part of health. The interactions between health, appearance, and well-being, while complex and beyond the scope of this article, create further difficult resource allocation decisions.

Many would argue that to avoid this decision, new resources should come into the oral health service from elsewhere or taxes should be raised to provide extra resources, but this simply defers the resource allocation problem to a wider context and a decision about whether this extra resource would have been better used elsewhere on other potential uses.

### The Remuneration of Dental Teams

Oral health services in most countries have remained at the periphery of general health services (Kandelman et al. 2012). In developing countries, access to oral health care is often restricted to general health clinics and hospitals, particularly for more deprived subpopulations and/or rural areas (Kandelman et al. 2012; Liu et al. 2016; Petersen et al. 2020). In developed countries and urban centers of developing countries, oral health care is principally delivered through freestanding clinics run as independent small businesses (Watt et al. 2019). While this small independent clinic approach to delivery of care is likely to have positive aspects, it presents specific issues when thinking about how dentistry is remunerated, given that sustainability or profitability of dental clinics will be a major objective of the provider.

Payment of dentists in the independent dentist setting has traditionally focused on fee-for-service (FFS) systems that pay according to activity with limited examples of capitation and small numbers of dental teams being remunerated by salary. The concept of pay for performance has become more widespread in general health recently but has not been employed widely in dentistry (Grytten 2017). Although there is limited evidence directly in oral health, evidence from other health settings suggests that providers respond to remuneration incentives, but it is not clear how much effect is due to measuring and informing about performance itself (Gosden et al. 2000). It is likely that due to the small business nature of dentistry, dental teams would be more likely to respond to these remuneration incentives (Brocklehurst et al. 2013). In particular, the predominant FFS model in dentistry tends to result in treatment being favored over provision of preventive care. Capitation-based services should theoretically result in less treatment and more prevention, as effective prevention reduces demands on the provider without reducing provider income, although evidence for primary care physicians did not support this (Gosden et al. 2000). In a pilot project switching some primary care dentists from FFS to capitation in Northern Ireland, treatment volumes fell but preventive interventions did not increase (Hill et al. 2017). Pay-for-performance models have been investigated for doctors in hospital settings with no impact found on patient outcomes, although quality of care did improve (Mathes et al. 2019).

Reforms of dental services now consider blended approaches to remuneration, but these are at an early stage and still under investigation (Listl et al. 2019). Such blended systems typically use a mix of different ways of remunerating dentists, such that dental professional may receive a capitation payment for their patients but also receive fee for service for specific interventions and pay for performance for achieving certain quality measures. Furthermore, payment systems are only one aspect of managing provider behavior, and changing behavior toward prevention will require consideration of other aspects.

## Which Economic Techniques and Principles Might Be Useful to Understand and Predict Impact of Reorientation?

Before considering the economic impact of reorienting health services toward prevention, it is important to consider which techniques might be useful in understanding this. Within this review, only a brief overview of relevant aspects of economic evaluation is considered (more detailed texts are available; e.g., Drummond et al. 2005).

The simplest economic technique is to consider the effect on cost alone (cost minimization analysis [CMA]). When calling for increased resources for prevention, the justification is often that this will be cost-saving as future treatment needs are reduced. However, evidence about the cost-saving nature of programs is often unclear in terms of where the initial investment will come from but also what happens to the savings. Often reducing treatment needs does not result in resources being released because those resources are then used in other ways (Listl et al. 2019).

Broader evaluation requires outcomes to be considered alongside costs. Health outcomes are often defined in disease-specific terms, but this limits comparability between programs addressing different diseases, and it is not always clear what impact the outcome has on a patient. Therefore, various measures have been developed for valuing health states such as the quality-adjusted life year (QALY) (Williams 1985). A corresponding measure, the quality-adjusted tooth year (QATY), has been proposed (Birch 1986). Both measures assume the value and duration of a health state are independent. The healthy year equivalent (HYE) relaxes this assumption (Mehrez and Gafni 1989). All these instruments measure the value of health independent of nonhealth aspects and in oral health may not be sensitive to small changes in the generic full health–immediate death scales used (Vernazza et al. 2012).

Taking a broader approach, willingness to pay (WTP) measures the maximum amount an individual is willing to forgo in monetary terms to gain a given health state improvement and allows nonhealth aspects of programs to be considered. One criticism of this measure is that WTP will be related to ability to pay, but it has been shown that this issue can be dealt with statistically (Donaldson 2001). The use of WTP, either derived through the method of contingent valuation or through discrete-choice experiments, has gained popularity in dentistry (Tan et al. 2017).

The economic techniques of cost-effectiveness analysis (CEA), cost-utility analysis (CUA), and cost-benefit analysis (CBA) use these health outcomes in combination with costs. However, with all of these techniques, where programs are deemed to be more costly but more effective, this leaves difficult choices about where the extra resource will come from.

For economic analyses to be useful, frameworks are required to allow decisions about the efficiency of investing in different combinations of programs to be made. Mathematical approaches such as integer programming may be used (Birch and Donaldson 1987), but these rely on having full economic information on all programs, which is rarely the case. Recognizing this, approaches have been developed to identify improvements in (rather than maximizing) efficiency (Birch and Gafni 1992).

While these approaches are useful in considering a single measure of health, very often, multiple, often competing, objectives need to be satisfied. The concepts of multicriteria decision analysis have therefore been adapted, with 1 common framework being program budgeting marginal analysis (PBMA) (Peacock et al. 2006). In this approach, various programs within a budget are selected by stakeholders for review against a set of weighted criteria. The final decisions recommend certain existing programs for disinvestment to allow investment in other programs. The approach is pragmatic and requires some subjective judgments but does allow multiple objectives to be satisfied (Peacock et al. 2006).

## What Is the Existing Evidence about the Economic Impact of Reorienting Health Services toward Prevention?

Given that most oral health services have not yet reoriented toward prevention, there is limited evidence about the economic impact of such changes. In addition, any such evidence is likely to be context specific, dependent on, for example, epidemiology, the value of oral health, workforce, existing health services, and wider determinants of access and health.

### Economic Analyses

Looking at costs alone, taking a within-program view, there are many examples of both cost-saving and cost-increasing preventive programs across health (Cohen et al. 2008). In dentistry, most reported evaluations increased costs, although the methodological rigor has been questioned (Kallestal et al. 2003). One example of a cost-saving intervention is the Childsmile program in Scotland (Anopa et al. 2015). In this national program involving supervised toothbrushing in the preschool setting, the measured dental treatment costs fell from £8.8 million to £4.8 million over the 8-y period of the program, while the annual cost of the program was £1.8 million. Although there could be concerns about whether all of the reduction in treatment costs is attributable to the program, taken at face value, the cost saving in year 8 was £2.2 million. However, there is no evidence that this reduced the overall expenditure on oral health care services, which may or may not have been an objective of the program but is often used as an argument to justify preventive expenditures.

In dental economic evaluations, most preventive programs had a positive effect on health or wider benefits, but this came at increased cost (Eow 2019). This leaves the difficult decisions that have already been discussed concerning which programs to invest in and where to reallocate resources from.

While this article is not intended to comprehensively review all of the economic evaluations of dental prevention interventions in detail (and readers are referred to the existing review by Eow [2019]) it is important to remember that the interventions are context specific, and any policy must take into account the most appropriate setting and issues around access to these interventions.

### Resource Allocation

Looking at broader resource allocation, one of the likely objectives, even in a multicriteria approach, is likely to be maximizing total benefit to the population or societal well-being. Given the discussions about measures of benefit, it is worthwhile briefly reviewing the evidence around WTP values elicited for prevention, as these should reflect societal benefit where measured properly. Overall, it seems that prevention is valued by society (Walshaw et al. 2019), but when treatment and prevention are directly compared, treatment and particularly those interventions that improve appearance are valued over prevention (Carr et al. 2018) or at similar levels (Tianviwat et al. 2008), although this may have been related to the uncertainty around need for treatment.

Undertaking resource allocation exercises has very rarely been done in dentistry. One example of integer programming was undertaken for children’s dental services in southern Thailand (Tianviwat et al. 2008). This was based on using parental WTP values as the measure of benefit, and to maximize these, allocating less resources to prevention (sealants) was recommended with extra resources moving to restorations. While PBMA has featured single dental programs in wider-scoped studies, only 2 have been undertaken directly in dentistry (Holmes et al. 2018; Vernazza et al. 2018). While Holmes et al. (2018) were unable to complete their PBMA due to system changes, early findings from Vernazza et al. (2019) suggest that prevention was not prioritized over current treatment due to its cost. The findings also suggest that the objectives of oral health services are often not explicit, and where they are, there are often multiple competing objectives.

These findings suggest that prevention is not valued highly by the public and policy makers, and a better case must be made. This may partly be due to cases often being presented that involve universal rather than targeted delivery. However, the case is unlikely to be successful if it relies on cost-saving arguments alone, and so wider measures of benefit, such as those illustrated by WTP, should be considered. Even where the case is clear, it may be that the objectives of a service are viewed differently by different stakeholder groups, and it is not clear who should have the greatest influence here.

A further issue for oral health is that budgets for individual-level services and population-based measures are often held separately and may be the responsibility of different managers or organizations. While reallocation within individual-level service budgets is considered here, there may be a case that the budget would be best reallocated to population-based measures, and the separation makes reallocation between these 2 aspects more difficult.

### Provider Remuneration

There is limited evidence in oral health of the potential effects of the newer concepts of pay for performance and blended payment systems. Previous discussions highlighted that incorporating some aspects of pay for performance, where health outcomes are rewarded, would be beneficial (Pitts et al. 2019). However, measuring health outcomes (e.g., caries avoided) and agreeing on relevant metrics is difficult, and often the simpler alternative of using process measures (e.g., number of patients receiving fluoride varnish) as the measure of performance is used. In addition, as oral health is dependent on wider societal determinants, measures of provider performance need to be adjusted for between-provider differences in baseline oral health and risks of disease among patients. It may be seen as unfair to penalize dental providers for baseline poor oral health of their patients. Both of these issues can be overcome with appropriate baseline information/adjustment and careful consideration of the measures used, but this will require further work.

## Conclusion and Recommended Actions

**Figure 2.**
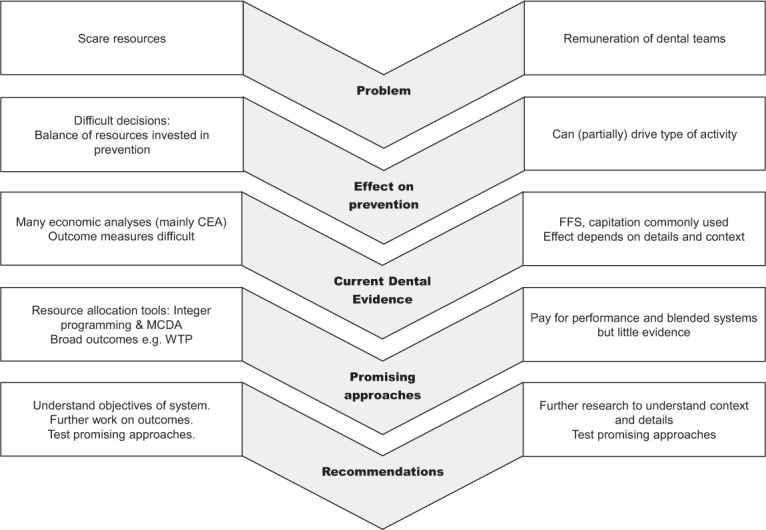
Summary of the 2 main issues covered in this article, existing evidence and recommendations. CEA, cost-effectiveness analysis; FFS, fee for service; MCDA, multicriteria decision analysis; WTP, willingness to pay.

A summary of the 2 main issues, evidence relating to dentistry and recommendations presented in this article, is presented in Figure 2. There is a strong desire for reorienting oral health services toward prevention and good evidence of which preventive interventions are effective. The economic impact is difficult to measure and wide-ranging but is important for making a case to policy makers, particularly those beyond oral health. The context of individual health services makes detailed recommendations difficult both for measuring the economic impact and in terms of recommended actions to reorient services.

Reorienting health systems is a complex task, but some broad recommendations can be made:

- In predicting and subsequently measuring economic impact, it is important to understand both the outcomes of any changes and the impact on resource requirements to produce those changes.
- In making a case to those outside of oral health, cost savings are unlikely to be relevant, and so it is important to also emphasize the outcomes and the value of health.
- The outcomes should be thought of in broad terms ideally measuring societal benefit (and also considering the long-term benefits and burdens across the life course).
- Resource reallocation is complex and should not rely solely on economic evaluations, and multiple stakeholders should be considered.
- In considering resource allocation, it is important to be clear about what the objectives of the oral health service are, although further debate is needed about who should set these (the public, patients, providers, and/or policy makers).
- Remuneration of dental providers is very dependent on context, but payments based on health outcomes would appear to be favorable, if difficult to measure.
- Although this article has focused on oral health services, it is vital to address wider determinants of oral health as well as engaging others in oral health. There is a complex interaction between changes to wider determinants and changes within oral health services, and this should be an area of further exploration.

## Author Contributions

C.R. Vernazza, contributed to conception, drafted and critically revised the manuscript; S. Birch, N.B. Pitts, contributed to conception, critically revised the manuscript. All authors gave final approval and agree to be accountable for all aspects of the work.
